# Comparison of Hydrocortisone Continuous Rate Infusion and Prednisolone or Dexamethasone Administration for Treatment of Acute Hypoadrenocortical (Addisonian) Crisis in Dogs

**DOI:** 10.3389/fvets.2021.818515

**Published:** 2022-01-25

**Authors:** Athanasia Mitropoulou, Mia-Katharina Häuser, Hendrik Lehmann, Katarina Hazuchova

**Affiliations:** Department of Veterinary Clinical Sciences, Small Animal Clinic, Justus-Liebig-University Giessen, Giessen, Germany

**Keywords:** Addisonian crisis, canine (dog), hydrocortisone (HC), DOCP, fludrocortisone, prednisolone, dexamethasone, hypoadrenocorticism

## Abstract

**Objectives:**

To determine whether administration of intravenous hydrocortisone is a safe and effective alternative treatment in comparison to the traditional treatment with prednisolone/dexamethasone in dogs presenting with Addisonian crisis; and to assess if there is any advantage of the former over the latter in normalisation of electrolyte imbalances and in hospitalisation length in these dogs.

**Methods:**

Medical records of client-owned dogs with hypoadrenocorticism were retrospectively reviewed. Time until normalisation of sodium and potassium concentration, intravenous fluid needs over the first 24 h and hospitalisation length were compared between hydrocortisone and prednisolone/dexamethasone treated dogs.

**Results:**

Twenty-five dogs met the inclusion criteria; 13 received hydrocortisone and 12 prednisolone/dexamethasone. Intravenous hydrocortisone was well-tolerated but failed to prove superiority in terms of time to normalisation of sodium and potassium concentration. Interestingly, potassium normalised in all dogs prior to discharge, but sodium did not in 1/11 hydrocortisone and 5/9 prednisolone/dexamethasone treated dogs with initial hyponatraemia (*p* = 0.05). Hydrocortisone treated dogs, however, had more electrolyte re-checks [hydrocortisone treated dogs, median (range): 4 (2–16); prednisolone/dexamethasone treated dogs: 2 (0–6); *p* = 0.001]. There was no difference in intravenous fluid needs over the first 24 h but hydrocortisone treated dogs had longer hospitalisation [hydrocortisone: 81 (45–309) h; prednisolone/dexamethasone: 52 (22–138) h; *p* = 0.01].

**Clinical Significance:**

Intravenous hydrocortisone is well-tolerated and safe, but no clear additional benefit over traditional glucocorticoid replacement could be identified. Also, it might result in longer hospitalisation time and more intensive monitoring.

## Introduction

Acute adrenocortical insufficiency or Addisonian crisis (AC) is a life-threatening condition that results from acute insufficiency of adrenocortical hormones (cortisol, aldosterone). Affected dogs typically present with signs of hypovolaemic shock, dehydration, and metabolic derangements (hyperkalaemia, hyponatraemia, metabolic acidosis, hypoglycaemia) (1). Unrecognised and left untreated, AC can be fatal, but with appropriate treatment the majority of dogs show fast recovery (within 24–48 h) and a good short- and long-term prognosis, with a median survival time of 4.7 years (1–3). Possible complications that may be encountered during treatment, include severe gastrointestinal ulceration and haemorrhage, disseminated intravascular coagulation, sepsis due to bacterial translocation from the gastrointestinal tract, and aspiration pneumonia due to megaoesophagus (3).

Although hypoadrenocorticism (HA) is an uncommon endocrine disease in dogs, several studies have reported on its clinical picture, diagnostic tests and long-term treatment of this condition (2, 4–7). On the other hand, treatment of AC, possible complications and outcome have mainly been described in book chapters or reviews (1, 3, 8, 9). The two cornerstones of treatment of AC are fluid resuscitation (to restore fluid volume and correct electrolyte and acid base imbalances), as well as administration of a rapidly acting source of glucocorticoids. Traditionally, dexamethasone and prednisolone have been used in the treatment of AC; the dosages varied based upon clinical experience rather than prospective or retrospective studies (3). In recent years, there have been some changes to the management of HA, including introduction of Zycortal® (Dechra Veterinary Products), a licenced veterinary product for mineralocorticoid replacement containing desoxycorticosterone pivalate (DOCP), and the use of hydrocortisone sodium succinate (HSS) as a continuous rate infusion (CRI) in the management of AC at some institutions (10). Hydrocortisone is a relatively cheap synthetic analogue of cortisol with glucocorticoid and mineralocorticoid activity identical to that of endogenous cortisol (11). It has been shown in experimental studies of healthy dogs and dogs with mitotane-induced hypocortisolaemia that administration of HSS as CRI produced a significant and sustained increase in plasma cortisol concentration (12, 13), reaching cortisol levels of dogs undergoing major abdominal, thoracic or orthopaedic surgery (14). Based on these findings, it has been suggested that HSS CRI should provide sufficient glucocorticoid and mineralocorticoid activity to treat AC (12). This could be confirmed by Gunn et al. (10), who used HSS CRI to treat AC in 30 dogs with newly diagnosed naturally-occurring spontaneous primary HA, and concluded that this treatment alongside IV fluid administration is well-tolerated and results in rapid control of hyperkalaemia. Side effects only occurred in one dog, who developed neurological signs most likely attributable to rapid increase in sodium concentration. Therefore, the authors recommended that caution should be taken to avoid rapid correction of sodium, particularly in patients with extreme hyponatraemia. This retrospective study only included patients treated with hydrocortisone CRI, preventing comparison to the “traditional” management using prednisolone/dexamethasone.

Although information about hydrocortisone use in animals with AC is scarce, in human medicine, administration of HSS as an initial bolus followed by a CRI is the mainstay of treatment of adrenocortical crisis in patients with primary HA (15–17). If hydrocortisone is unavailable, prednisolone is suggested as an alternative, while dexamethasone should only be given if no other glucocorticoid is available (15).

The aim of this study was, firstly, to determine whether administration of hydrocortisone CRI is a safe and effective alternative treatment compared to prednisolone/dexamethasone in dogs presenting in AC. The second aim was to assess if there is any advantage of hydrocortisone CRI over prednisolone/dexamethasone in normalisation of electrolyte imbalances and in hospitalisation length in dogs presenting in AC.

## Materials and Methods

### Case Selection

Medical records of client-owned dogs with naturally-occurring confirmed primary HA admitted to the Small Animal Clinic of Justus-Liebig- University of Giessen, Germany, from January 2013 to July 2021 were retrospectively reviewed. Inclusion criteria were a confirmed glucocorticoid and mineralocorticoid deficiency as proven by an inadequate cortisol production during an ACTH stimulation test, as well as presentation with acute AC. Acute AC was defined as a life-threatening emergency due to glucocorticoid and mineralocorticoid insufficiency, clinically manifested as hypotension, dehydration, electrolyte (hyperkalaemia and/or hyponatraemia) and acid-base abnormalities (1). Cases with findings compatible with AC but a history of recent glucocorticoid administration were evaluated on an individual basis and only included in the study if the dose and frequency of administration of the glucocorticoid preparation was unlikely to suppress hypothalamus-pituitary-axis and/or interfere with cortisol measurement (e.g., dogs that received a single dexamethasone injection). Exclusion criteria were incomplete medical records leading to inadequate documentation of the treatment protocol and disease course; patients presenting with HA but not with AC; patients presenting with atypical HA or patients that presented with AC and received a combination of dexamethasone/prednisolone bolus followed by HSS CRI.

### Demographic, Clinical, and Laboratory Data

Demographic data including age, sex, reproductive status, weight, and breed; history (including previous treatment and any known previous illness); physical examination findings; systolic blood pressure (SBP) measurement and clinicopathological data were recorded. Blood pressure measurements were obtained using either Doppler sphygmomanometry or oscillometric method on a peripheral limb utilising a cuff size that was ~40% of the limb diameter. Blood was collected from a peripheral vein into EDTA or heparinised tubes for haematological (ADVIA 2120; Siemens Healthcare GmbH; or ProCyte Dx, Haematology Analyzer (IDEXX) if presented out of hours) and biochemical (ABX Pentra C400; Horiba ABX SAS) analysis, respectively. Venous blood gas analyses (including electrolytes and lactate) were carried out with blood collected into pre-heparinised syringes or heparinised tubes and measured using cobas b 221 POC system (Roche Diagnostics GmbH) (until February 2020) or cobas 123 POC system (from February 2020). Blood for serum cortisol measurements were collected before and 1 h after IV administration of tetracosactide (Synacthen 0.25 mg/ml Solution for Injection; Alfasigma S.p.A.). Tetracosactide was administered IV at a dose 5 μg/kg, or at a dose of 250 μg/dog in dogs weighing >15 kg and 125 μg/dog in dogs weighing <15 kg. Cortisol concentrations were measured using a chemiluminescent assay (Centaur XP, Siemens Medical Solutions) at an external laboratory (Bioscientia Healthcare GmbH).

### Treatment and Monitoring

Intravenous fluid treatment was started immediately after peripheral vein catheterisation and collection of blood samples. Traditional resuscitation endpoints [normalisation of clinical perfusion parameters (CRT, heart rate, pulse quality) or/and attainment of SBP > 90 mmHg] were reached with the use of crystalloids (normal saline or balanced isotonic solution) as boluses followed by CRI. The type and rate of IV fluids as well as the frequency of clinical and clinicopathological monitoring were chosen by the attending clinician. After performing the ACTH stimulation test, dogs presented before December 2017 received prednisolone IV or PO (Solu-decortin H, Merck; Prednitab vet, CP-Pharma) at an initial dose of 0.1–1 mg/kg, or dexamethasone IV (Hexadreson, MSD) at an initial dose of 0.1–1 mg/kg; this group of dogs is named “traditional treatment (TT) group” throughout this article. Dogs presented from December 2017 onwards received HSS (Hydrocortisone, Pfizer Sine) CRI at a dose of 0.25 or 0.5 mg/kg/h (further named “HSS group”), which was continued until the dog was considered clinically stable and the potassium concentration dropped below 5 mmol/L. At this timepoint, HSS CRI was stopped in most dogs and oral prednisolone was administered. However, some clinicians chose to continue HSS CRI even after normalisation of the clinical picture and potassium concentration until the results of the ACTH stimulation test became available. Mineralocorticoid treatment with fludrocortisone (Astonin H, Merck; starting dose 0.02 mg/kg/day PO) or DOCP (Zycortal, Dechra; starting dose 1.5–2 mg/kg SQ) was started once the diagnosis was confirmed or, in cases with high level of clinical suspicion for HA, even before receiving the results of the ACTH stimulation test, once the dog was clinically stable and well-hydrated (in DOCP treated dogs) and with voluntary food intake (in fludrocortisone treated patients).

Dogs were discharged with a combination of fludrocortisone/DOCP and oral prednisolone once clinically unremarkable, eating, and the intravenous fluid therapy had ceased.

### Outcome Parameters

Following variables were compared between the groups (TT vs. HSS group): proportion of dogs that achieved normalisation of sodium and potassium concentration; time until normalisation of sodium and potassium; sodium and potassium concentration at the time of normalisation; number of re-checks of sodium and potassium concentration during hospitalisation; volume of IV fluids administered within the first 24 h of hospitalisation; survival to discharge; hospitalisation length. Furthermore, time until normalisation of potassium was compared with the time to achieve sodium normalisation within the HSS and within the TT group. In this study, hyponatraemia was defined as sodium concentration <140 mmol/L and hyperkalaemia was defined as potassium concentration > 5 mmol/L. The time until normalisation of sodium and/or potassium concentration was calculated in hours from the start of the HSS CRI or administration of prednisolone/dexamethasone until the time point of normalisation of each electrolyte (Na ≥ 140 mmol/L, K ≤ 5 mmol/L). If sodium or potassium were normal upon admission or did not normalise until discharge, these dogs were omitted from the comparison of the time until normalisation of sodium or potassium.

Finally, to enable comparison of severity of illness between the TT and HSS group, shock index (SI) and the fast Canine Acute Patient Physiologic and Laboratory Evaluation (APPLE_fast_) score were calculated retrospectively from parameters obtained upon presentation. Where SBP was documented upon presentation, SI was calculated as heart rate/SBP, with values > 1 indicating moderate to severe shock (18). The APPLE_fast_ score was calculated including the mentation score at presentation as well as the most abnormal value identified over the 24 h period following admission of the following four parameters: glucose, albumin, lactate, platelet count (19). This 5-variable model with a maximum score of 50 (50 indicating the most severe illness) allows a stratification of the mortality risk in hospitalised dogs.

### Statistical Analysis

Statistical analysis was performed using a statistical software (IBM SPSS Statistics for Windows, version 26, IBM). Graphs were made using GraphPad Prism for Windows version 8.0 (GraphPad Software). Continuous data were tested for normality using visual evaluation of histograms and the Kolmogorov-Smirnov Test. Normally distributed data are presented as mean ± standard deviation (SD) and non-normally distributed data are presented as median (range). Data are presented as median (range) if the assessed variable was non-normally distributed in one of the groups. Normally distributed variables were compared using Student's *t*-test and non-normally distributed data were compared using the Mann-Whitney-U test. Categorical data were compared using Fisher's exact test. Statistical significance was set at *p* < 0.05.

## Results

### Demographic, Clinical, and Laboratory Data

A total of 49 cases were identified. Twenty-four cases were excluded from the study for following reasons: missing files (1/49, 2%), presentation with primary HA without AC (7/49, 14.2%), presentation with atypical primary HA (9/49, 18.4%) or secondary HA (1/49, 2%), deviation from the standard protocol of care (4/49, 8.2%). Additionally, one dog was excluded as it was discharged at owner request against veterinary advice and still in critical condition, to continue treatment in another facility (1/49, 2%). Finally, another dog was excluded due to serious comorbidities (1/49, 2%), precluding assessment of treatment success. Ultimately, 25 dogs met the inclusion criteria, of which 13 (52%) received HSS CRI (HSS group) and 12 (48%) received traditional therapy with prednisolone/dexamethasone (TT group). In the HSS group, there were eight mixed breed and five purebred dogs (one of each: Boerbel, German Pincher, Border Collie, German Jagdterrier, Magyar Viszla). In the TT group, nine dogs were purebred (two Jack Russel Terriers and one of each: Bernese Mountain Dog, Boston Terrier, Chihuahua, Doberman Pincher, Havanese, Pyrenean Mountain Dog, Rottweiler) and three were mixed breed. There was no difference in age or body weight between treatment groups, but more female dogs were treated with HSS than with TT (see Table 1). Of the 11 female dogs in the HSS group, eight were spayed; both male dogs in the HSS group were intact. All four female dogs in the TT group were spayed, while 4/8 male dogs were intact. Furthermore, there was no difference in vital parameters, SBPor disease severity based on SI and Apple_fast_ score (Table 1). There was no difference between the treatment groups in duration of clinical signs prior to presentation (Table 1). The proportion of dogs presented out of hours was similar, and there was also no difference in the proportion of dogs pre-treated with corticosteroids prior to presentation or dogs showing melaena (Table 1).

**Table 1:** Comparison of baseline characteristics including signalement, history, physical examination, and illness severity scores between dogs treated with hydrocortisone CRI (HSS group; *n* = 13) and dogs treated with prednisolone/dexamethasone (TT group; *n* = 12).

**Variable**	**Treatment Group**		***p*-value**
	**HSS**	**TT**	
Number of dogs	13	12	–
Age (years)	5.4 (±3.1)	5.6 (±2)	0.83
Gender	2 M	8 M	0.02
	11 F	4 F	
Body weight (kg)	18.6 (4.7–50)	16.5 (4.4–65.0)	0.69
Respiratory rate (breaths/min)	28 (20–40)	28 (22–44)	0.50
Heart rate (beats/min)	109 (±37)	104 (±27)	0.71
Temperature (°C)	38.2 (±0.5)	37.7 (±0.8)	0.07
Blood pressure (mmHg)	103 (±18)	102 (±33)	0.89
Shock index (SI)	1.06 (±0.45)	1.15 (±0.67)	0.74
Apple_fast_ score	20.3 (±4.5)	20.3 (±5.9)	0.99
Presentation out of hours	4/13	4/12	1
Duration of symptoms prior presentation (days)	6 (3–150)	5.5 (1–69)	0.89
Melaena	0/13	4/12	0.39
Treatment with steroids prior to presentation	5/13	3/12	0.67

*Significance at p < 0.05*.

*CRI, continuous rate infusion; M, male; F, female*.

Baseline clinicopathological variables, including blood gas analysis results and electrolyte concentration, are presented in Table 2. There was no significant difference between the groups for any of these parameters, including electrolyte concentrations.

**Table 2:** Comparison of the baseline clinicopathological variables including electrolytes and blood gas analyses values between dogs treated with hydrocortisone CRI (HSS group; *n* = 13) and dogs treated with prednisolone/dexamethasone (TT group; *n* = 12).

	**Parameter**	**HSS-group**	**TT-group**	***p*-value**
**CBC**
	Number of patients (*N*)	13/13	12/12	
	Haematocrit (%)	41.9 (±15.3)	41.4 (±17)	0.93
	Neutrophils (G/L)	8.9 (1.6–61.5)	6.4 (4.7–17.8)	0.57
**Biochemistry**
	Number of patients (N)	13/13	12/12	
	Urea (mmol/L)	18.9 (±8.5)	23.4 (±12.3)	0.31
	Creatinine (μmol/L)	160.1 (±68.9)	199.5 (±121.4)	0.34
	Albumin (g/L)	28.7 (±4.1)	27 (±5.6)	0.41
	Phosphor (mmol/L)	2.1 (±0.4)	2.1 (±0.7)	0.81
	Number of patients (N)	13/13	10/12	
	c-reactive protein (mg/dL)	56.3 (±37.9)	67.6 (±62.8)	0.64
**Blood gas analysis**
	Number of patients (*N*)	13/13	11/12	
	pH	7.27 (±0.05)	7.31 (±0.05)	0.14
	pCO2 (mmHg)	37.3 (±6.2)	35.1 (±7.9)	0.46
	HCO3 (mmol/L)	16.7 (±3.3)	18 (±3.7)	0.37
	BE (mmol/L)	−10.8 [−12.5–(−2.6)]	−7 [−13.3–(+2.3)]	0.39
**Electrolytes**
	Number of patients (*N*)	13/13	12/12	
	Na (mmol/L)	133.1 (±6.5)	127.4 (±8.2)	0.06
	K (mmol/L)	7 (±1.3)	6.5 (±1.3)	0.34
	Cl (mmol/L)	101.8 (±8.7)	98.7 (±10.1)	0.42
	iCa (mmol/L)	1.39 (±0.34)	1.25 (±0.13)	0.19
	Glu (mmol/L)	5.3 (±2)	5 (±1.4)	0.62
	Number of patients (*N*)	13/13	8/12	
	Lactate (mmol/L)	1.7 (0.7–7.2)	2.1 (0.3–3.3)	0.92

*Significance at p < 0.05*.

### Treatment

All dogs received intravenous fluids before starting one of the two different protocols. The choice of resuscitation and maintenance fluid type was left to the discretion of the attending clinician. The majority of patients received either balanced electrolyte solution (11/25; Sterofundin Iso, B. Braun) or normal saline (14/25; NaCl 0.9%, B.Braun) as resuscitation fluid. Normal saline, balanced electrolyte solution or isonatraemic fluid (prepared by mixing 450 ml of 5% glucose (G5, B.Braun) with 50 ml of 8.4% sodium hydrogen carbonate (NaBiC 8.4%, B. Braun) were used as maintenance fluids. Changes of the fluid type were guided by electrolyte measurements in individual patients.

There was no significant difference in the length of fluid treatment before starting HSS CRI or prednisolone/dexamethasone administration to the TT group (HSS group: 3 (1–8) h; TT group: 3.5 (2–17) h; *p* = 0.35). The HSS CRI was started after 5 (1–13) h, while in the TT group prednisolone/dexamethasone was administered after 5.75 (3–18) h (*p* = 0.23) after presentation. In the HSS group, eight dogs were treated with an HSS CRI dose of 0.5 mg/kg/h and five with a dose of 0.25 mg/kg/h. The HSS group dogs remained on the HSS CRI for a median of 31 (5–103) h before switching to oral prednisolone (Prednitab vet, CP-Pharma). In the TT group, six dogs received IV prednisolone (Solu-decortin H, Merck), two received IV dexamethasone (Hexadreson, MSD) and four were treated with oral prednisolone (Prednitab vet, CP-Pharma) from the start, without any previous administration of parenteral glucocorticoids.

In terms of mineralocorticoid treatment, DOCP or fludrocortisone were administered to dogs in both groups. However, in the HSS group, there was a significant delay in DOCP/fludrocortisone administration in comparison to the TT group [HSS group: 30 (4.5–163) h; TT group: 6 (3–18) h; *p* = 0.001]. As HSS does have mineralocorticoid effects, when the timepoint of HSS CRI start was considered to be the start of mineralocorticoid, there was no difference in the mineralocorticoid treatment start between the groups [HSS CRI start after 5 (1–13) h vs. DOCP/fludrocortisone administration in the TT group after 6 (3–18) h; *p* = 0.19].

### Outcome Parameters

No difference could be detected between the two groups regarding the volume of intravenous fluids the dogs received in the first 24 h after presentation [HSS group: 123.7 (97.8–245.7) ml/kg; TT group: 149.5 (44–353.4) ml/kg; *p* = 0.72].

**Figure 1:**
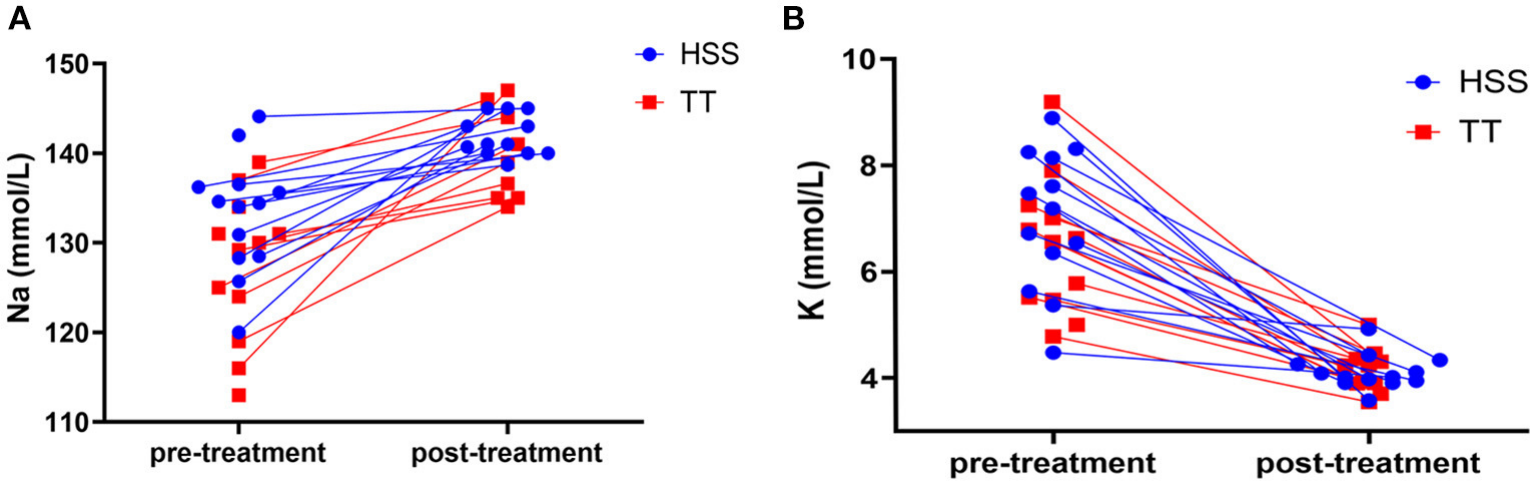
Sodium **(A)** and potassium **(B)** concentration in individual dogs before and after treatment with HSS CRI (HSS group; blue dots) and prednisolone/dexamethasone (TT group; red rectangles).

**Figure 2:**
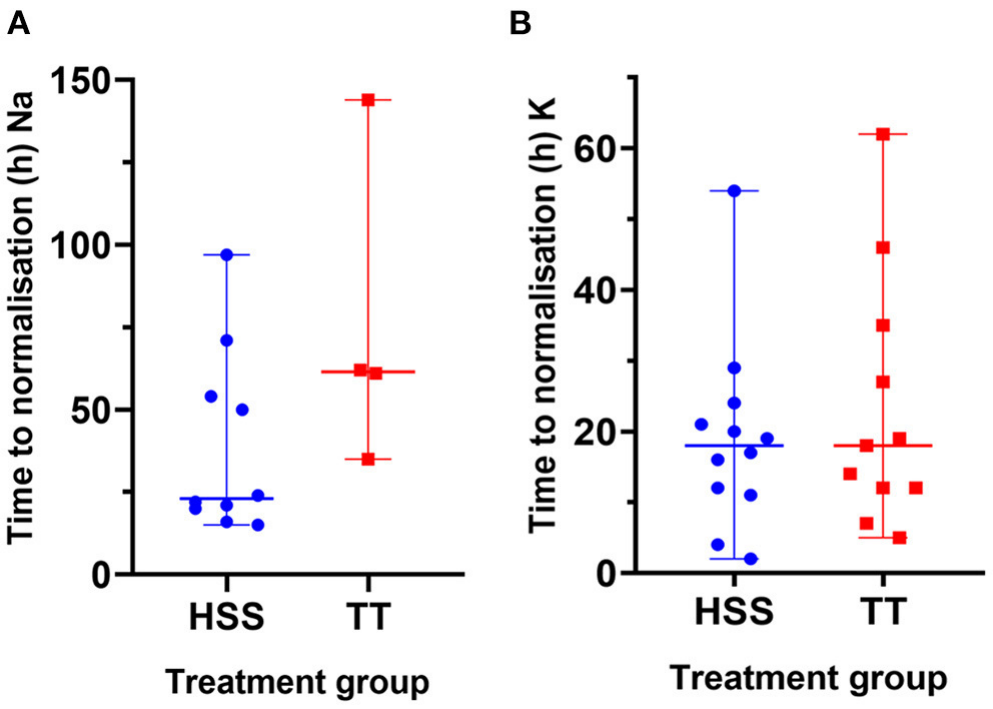
Comparison of time to normalisation of sodium **(A)** and potassium **(B)** concentration between the HSS (blue dots) and TT group (red rectangles). Horizontal line represents median, whiskers represent range.

There was no difference between the HSS and TT group in pre- or post-treatment sodium or potassium concentration (Figure 1; Table 3) or time to achieve normalisation of sodium or potassium concentration in dogs with initial electrolyte abnormalities (Figure 2; Table 3). However, within the TT group, potassium normalised significantly faster than sodium [K: 16 h (5–62); Na: 61.5 h (35–144); *p* = 0.017]. No such difference could be detected within the HSS group [K: 18 h (2–54); Na: 23 h (15–97); *p* = 0.052].

**Table 3:** Comparison of pre- and post-treatment sodium and potassium concentration as well as time until concentration normalisation of these two electrolytes between dogs treated with hydrocortisone CRI (HSS group; *n* = 13) and dogs treated with prednisolone/dexamethasone (TT group; *n* = 12).

	**HSS-group**	**TT-group**	***p*-value**
**Sodium**			
Na pre-treatment (mmol/L) (all dogs)	133.1 (±6.5) (*n* = 13)	127.4 (±8.2) (*n* = 12)	0.06
Na post-treatment (mmol/L) (all dogs with re-check Na available)	141 (138.7–150.5) (*n* = 13)	139 (134–147) (*n* = 9)	0.23
Number of dogs with Na <140 mmol/L pre-treatment	11/13	12/12	0.48
Number of dogs with Na ≥ 140 mmol/L post-treatment (only dogs with initially low Na included)	10/11	4/9	0.05
Time until Na normalisation in hours (only dogs with initially low Na included)	23 (15–97)	61.5 (35–144)	0.11
**Potassium**			
K pre-treatment (mmol/L) (all dogs)	7 (±1.3)	6.5 (±1.3)	0.336
K post-treatment (mmol/L) (all dogs)	4.1 (±0.3)	4.1 (±0.4)	0.84
Number of dogs with pre-treatment *K* > 5 mmol/L	12/13	11/12	1
Number of dogs with post-treatment *K* ≤ 5 mmol/L (only dogs with initially high *K* included)	12/12	11/11	1
Time until *K* normalisation in hours (only dogs with initially high K included)	19.1 (±13.5)	23.4 (±17.8)	0.53

*Significance at p < 0.05*.

There was no difference in the proportion of dogs with hyperkalaemia between treatment groups prior to treatment start (all but one dog in each group had K > 5 mmol/l), and in all hyperkalaemic dogs potassium levels normalised prior to discharge or, in one dog in the TT group, prior to euthanasia (Table 3). This included eight dogs treated with an HSS CRI dose of 0.5 mg/kg/h and four administered a dose of 0.25 mg/kg/h.

All dogs in the TT group and 11/13 dogs in the HSS group were hyponatraemic upon presentation. Although not statistically significant, a numerical difference could be seen in the proportion of dogs that achieved normal sodium concentration post-treatment (Table 3). While 10/11 dogs of the HSS group with initially low sodium achieved normalisation, one dog remained hyponatraemic at the final re-check prior to discharge. This single dog received HSS CRI dose of 0.5 mg/kg/h, similarly to five of the dogs who achieved sodium normalisation; the five remaining dogs received HSS CRI of 0.25 mg/kg/h. In the TT group, sodium normalised in 4/9 dogs for which follow-up sodium concentration was available; 5/9 remained hyponatraemic and in the remaining three dogs of this group sodium was not re-assessed (*n* = 2) or the dog was euthanised before treatment response could be adequately evaluated (*n* = 1). Generally, however, less electrolyte re-checks were performed in TT group [2 (0–6)] during hospitalisation than in the HSS group [4 (2–16)] (*p* = 0.001).

All but one dog (included in the TT group) survived to discharge. This dog developed severe anaemia due to gastrointestinal bleeding and suffered cardiopulmonary arrest. Although it could be successfully resuscitated, the owner opted for euthanasia due to financial constraints. In all surviving dogs the treatment was well-tolerated. No neurological signs were observed with either of the HSS CRI dosages (0.5 vs. 0.25 mg/kg/h). There was a significant difference in the hospitalisation length between the two groups, with longer hospitalisation time in the HSS group [81 (45–309) vs. 52 (22–138) h; *p* = 0.01].

## Discussion

The results of this retrospective study indicate that hydrocortisone CRI is a safe and effective alternative to prednisolone/dexamethasone for treatment of AC. Both sodium and potassium normalised in all but one HSS-treated dog with mild hyponatraemia at discharge, irrespective of the administered HSS CRI dose (0.5 mg/kg/h and 0.25 mg/kg/h). In contrast, in some of the dogs treated with prednisolone/dexamethasone sodium concentration did not normalise during hospitalisation. However, a clear, clinically relevant advantage of HSS CRI over “traditional treatment” could not be demonstrated in this retrospective study.

The present study failed to detect a significant difference between the HSS CRI and prednisolone/dexamethasone group regarding the time to achieve normalisation of sodium or potassium concentration in dogs with initial electrolyte abnormalities. Potassium concentration generally normalised within less than a day in all dogs with initial hyperkalaemia, irrespective of the treatment group. Concerning sodium, more HSS-treated than prednisolone/dexamethasone-treated dogs achieved normalisation of sodium concentration prior to discharge, but the difference was not significant. Furthermore, although again not significant, faster normalisation of sodium concentration was achieved in HSS-treated dogs. Although these results suggest some potential benefit of HSS CRI at least regarding its effect on sodium concentration, a clear prove could not be obtained in this study. The reasons for this might be two-fold. There truly might be no advantage of HSS CRI over prednisolone/dexamethasone in treating AC in dogs. On the other hand, it is also possible that this study was unable to detect any difference between the treatment groups because of the small sample size and lack of standardisation of the frequency of monitoring. We have retrospectively calculated the number of cases needed to reach statistical significance when comparing the proportion of dogs reaching normal sodium concentration between the groups (20). Based on the proportion of dogs that reached normal sodium concentration in this study, the current study had an ~70% power to detect difference between the treatment groups. To achieve 80% power at 95 or 90% confidence, 12 or 10 dogs per group would be needed, respectively. Therefore, our study was underpowered and a few more dogs would have been needed to achieve significance.

The frequency of electrolyte re-checks in this study was based on the attending clinician's discretion. Importantly, fewer electrolyte re-checks were performed in the TT group in comparison to the HSS group. The shorter hospitalisation time of the TT group might also not have been sufficient for sodium normalisation. In the HSS group, sodium concentration normalised within 24 h in most dogs but in four dogs this took up to 4 days. However, the majority (8/12) of the TT group dogs were hospitalised for 2 days and only one dog was hospitalised for more than 4 days.

As mentioned above, an interesting finding of this study is that potassium concentration normalised within 24 h in the majority of dogs, irrespective of the treatment group, but this was not the case for sodium concentration. Although sodium concentration normalised around the same time as potassium within the HSS group, in the TT group, sodium normalisation took significantly longer. One of the mechanisms contributing to hyperkalaemia in dogs with AC, besides mineralocorticoid deficiency, is hypovolaemia and reduced renal perfusion, leading to reduced glomerular filtration rate (GFR) (3). Therefore, the fast normalisation of potassium, which in some dogs occurred before any mineralocorticoids were administered, might be attributable to the fluid treatment and restoration of renal perfusion and GFR. On the other hand, hyponatraemia in dogs with HA is caused by lack of aldosterone, resulting in impaired ability to conserve sodium in the distal nephron, but glucocorticoid deficiency might also contribute (secondary to stimulation of ADH secretion caused by lack of negative feedback of cortisol on paraventricular nucleus) (3). Therefore, mineralocorticoid alongside with glucocorticoid supplementation is needed to achieve normalisation of sodium concentration. This treatment needs longer time to exhibit its full effect in comparison to resolution of hypovolaemia and restoration of GFR, which plays a role in normalisation of potassium concentration, and with appropriate IV fluid treatment can be achieved faster. The finding that sodium normalised around the same time as potassium in the HSS but not in the TT group suggests that this outcome might be attributable to the mineralocorticoid effect of hydrocortisone. This, generally, is stronger than mineralocorticoid effect of prednisolone (3). However, as discussed previously, the higher frequency of monitoring in HSS treated dogs might also have played a role, allowing earlier detection of sodium normalisation in the HSS group. If this potentially faster sodium normalisation with HSS administration has any clinical implications cannot be established based on retrospective data.

The use of HSS CRI for treatment of canine AC has previously been reported in only one study (10), while administration of hydrocortisone (bolus followed by a CRI) is the current mainstay of treatment of human AC (15). In humans, hydrocortisone is clearly preferred over prednisolone and dexamethasone should only be given if no other glucocorticoid is available (15). This seems logical given the structural identity of hydrocortisone and endogenous cortisol (11). However, interestingly, even in human medicine there is little evidence suggestive of superiority of hydrocortisone over prednisolone in treating patients presented with AC. The only available evidence substantiating the choice of the glucocorticoid preparation originates from long-term studies of human patients with HA. In long-term patients, superiority of oral hydrocortisone over prednisolone in terms of mortality, cardiometabolic changes, and bone density, has been shown in several studies (21–24). Moreover, hydrocortisone is less potent and generally has fewer adverse effects than other synthetic glucocorticoids, and post-dosage levels can be easily measured in urine or saliva and the dosage adjusted accordingly (22).

As already mentioned, HSS CRI has only been evaluated in one study of dogs with AC, where side effects occurred in only one dog due to a too rapid correction of hyponatraemia (10). In terms of the other glucocorticoid preparations, long term side effects have been well-described in dogs with HA (2, 25). Although not specifically evaluated in dogs with AC, an increased risk for gastrointestinal bleeding or even perforation in dogs with hypotension or neurological disorders has been described in association with dexamethasone administration (26–28). Only two dogs in the present study received dexamethasone, and no side effects occurred. The only non-survivor in this study who experienced severe gastrointestinal bleeding had not received dexamethasone prior to referral or at our institution; this dog received IV prednisolone.

Similar to the situation in veterinary medicine, no systematic dose-response-studies have been performed to evaluate different glucocorticoid dosages for treatment of AC in humans. Therefore, the recommendations regarding glucocorticoid dosages for treatment of AC, including that of hydrocortisone, have been largely made on empirical basis (15). As this canine study was retrospective, it was not possible to evaluate why a particular HSS CRI dose (0.25 vs. 0.5 mg/kg/h) was chosen in individual dogs. Some clinicians might have chosen lower dose in dogs with severe hyponatraemia in an attempt to try avoid a too rapid increase of sodium concentration. Hypernatraemia as a result of HSS treatment was only documented in one dog in this study, treated with the higher HSS CRI dose. In this dog, an initially normal sodium concentration (142 mmol/L) increased to 150.5 mmol/L within 12 h, but no adverse effects occurred. The small number of dogs included in the HSS CRI group in this study precluded direct comparison of different outcome parameters (e.g., time until normalisation of electrolytes' concentration) between dogs treated with HSS CRI dose of 0.25 and 0.5 mg/kg/h. Therefore, no recommendations can be made on the HSS CRI starting dose from this study. A hydrocortisone CRI dose of 0.25 mg/kg/h, however, seems effective and is cheaper. A prospective randomised study with clearly defined monitoring scheme is needed to establish optimal dose of HSS CRI or even the need for an HSS bolus before starting the CRI for the treatment of dogs presenting with AC. Furthermore, in human medicine, besides hydrocortisone CRI, bolus therapy using the same hydrocortisone dose split into three to four intravenous or intramuscular injections might be used to treat AC (29). Therefore, intramuscular HSS treatment protocol might be considered for future studies in dogs.

One of hydrocortisone's advantages is its equal glucocorticoid and mineralocorticoid activity due to its ability to stimulate both glucocorticoid and mineralocorticoid receptors (11). Human patients presented with AC are treated with HSS CRI for the first 24 h and, if clinically stable, the dose is tapered over the next 48–72 h to the usual maintenance dose. Mineralocorticoid administration is only restarted when the total daily hydrocortisone dose is <50 mg (29). Given these above-mentioned properties of hydrocortisone, some clinicians at our institution chose to continue HSS CRI until the results of the ACTH stimulation test became available. This resulted in significant difference between the two groups regarding the timepoint of DOCP/fludrocortisone administration. Even though administration of one DOCP dose in dogs without HA is not harmful and the drug therefore can be given in suspected cases pending the ACTH stimulation test results, a matter of cost arises as DOCP is expensive in comparison to HSS (5). The cost of fludrocortisone is significantly lower, however in the recent years, there were some problems with its supply. Importantly, the use in dogs is off-label. When the timepoint of HSS CRI start was considered the start of mineralocorticoid administration in the HSS group, there was no difference in the mineralocorticoid treatment start between the groups.

In the present study, dogs treated with HSS CRI were hospitalised for significantly longer time periods compared with the dogs in the TT group. This might reflect the increasing standards of care and intensity of monitoring over the past years, especially regarding hospitalisation in the intensive care unit. All dogs included in the TT group presented before December 2017, while all HSS treated dogs presented after this timepoint. However, it is also possible that treatment with HSS CRI is generally associated with longer hospital stay and therefore also higher costs. This cannot be ascertained from a retrospective study, but the possibly higher costs should be born in mind when discussing treatment options with the client, especially when financial constraints exist.

This study has several limitations. The main limitation is its retrospective nature, resulting in lack of standardisation of clinical and laboratory reassessment timepoints in individual dogs. Some data was missing in individual patients and in some dogs very few electrolyte re-checks were performed, especially in those presented before 2017. Another drawback is the difference in time, all dogs in the TT group presented before 2017, while HSS treated dogs presented afterwards. This might have resulted in different standards of care applied to dogs receiving the two treatment protocols compared here and might have affected the hospitalisation length. Because of the retrospective nature of the study possible influencing factors in individual cases also cannot be excluded. This might include pre-treatment at the referring veterinarian (e.g., fluid therapy) in individual patients, but also individual differences in disease predisposition and pathogenesis. Although an immune-mediated destruction of the adrenal cortex is assumed to be the most common cause of HA in dogs, the pathogenesis might differ in different breeds as well as within individuals of one breed (30), similar to what has been proposed for other diseases with genetic background such as diabetes mellitus (31, 32). Therefore, although the clinical presentation of adrenocortical crisis might be very similar in all Addisonian patients (see definition in section Materials and Methods), the pathogenesis of the disease might be quite heterogenous and might have affected treatment outcomes in individual dogs. However, given its low prevalence, information on individual risk factors of HA is very limited and cannot be adequately addressed from a retrospective study. Another limitation of the study is that treatment decisions in each case were made by different clinicians with subjective judgements individualised to each case. However, as this was a retrospective review of medical records, the authors did not influence any of the decisions taken during treatment of the included dogs. Furthermore, the study cohort is small and, besides dogs with HA not presented in crisis, some dogs presented with AC had to be excluded because of missing files or deviation from the standard protocol of care. Because HA is a rare disease, multicentre studies might be better suited for evaluation of management of this condition. In such studies attention needs to be paid for participants to strictly comply with study protocol to prevent introduction of bias due to differences in standards of care between institutions. Another limitation is that some dogs had received glucocorticoids before presentation at our institution, which might have affected treatment response. However, there was no difference between the two groups in proportion of dogs that received glucocorticoids prior to referral. Moreover, based on the expected duration of action of the glucocorticoid preparations administered prior to admission and the route of administration, it is unlikely that substantial residual glucocorticoid activity was present in these dogs. Also, the effect of fluid treatment on electrolyte concentration, which was administered to all dogs prior to application of HSS or prednisolone/dexamethasone could not be evaluated retrospectively. Furthermore, in a retrospective study it is not possible to definitely rule out the presence of concurrent disorders, which might have affected outcome (especially electrolyte concentration). However, most dogs received a fairly standardised work-up including abdominal ultrasound and thoracic radiographs and no concurrent conditions were identified when reviewing the medical records.

Although the study cohort is small, and the study was underpowered to detect significant differences in the outcome parameters, it provides important information for the design of future prospective studies. A future prospective randomised study should include at least 12 dogs per group and regular assessments of electrolyte concentrations should be planed (ideally at least every 4–6 h). Although the current study did include 12 and 13 dogs per group, respectively, not all animals had hyponatraemia upon admission and in some dogs follow-up measurements were missing. Therefore, the actual sample size to assess changes in electrolyte concentrations (particularly sodium) was smaller. Based on the results of this retrospective study, it is expected that in an adequately powered prospective study HSS-treated dogs will experience faster recovery of sodium concentration. It is to be evaluated in a prospective fashion what clinical consequences will be associated with this faster sodium normalisation. Based on physiological principles, one might for example expect faster resolution of polyuria/polydipsia and better fluid conservation in the body. This, however, only can be evaluated in a prospective study.

In conclusion, the results of this study suggest that hydrocortisone CRI is well-tolerated and safe to use in patients presenting with AC, but no clear additional benefit over traditional glucocorticoid replacement using prednisolone/dexamethasone could be identified. Although HSS CRI is an effective alternative to the traditional treatment of AC, it might result in longer hospitalisation time, more intensive monitoring increasing staff workload and higher costs of hospitalisation.

## Data Availability Statement

The original contributions presented in the study are included in the article, further inquiries can be directed to the corresponding author.

## Author Contributions

AM collected and analysed data and wrote the manuscript. M-KH collected and analysed data. HL analysed data and edited the manuscript. KH conceived the study, analysed data, and edited the manuscript. All authors contributed to read and approved the final manuscript.

## Funding

This work was funded by the Open Access publication fund of the Justus-Liebig University.

## Conflict of Interest

The authors declare that the research was conducted in the absence of any commercial or financial relationships that could be construed as a potential conflict of interest.

## Publisher's Note

All claims expressed in this article are solely those of the authors and do not necessarily represent those of their affiliated organizations, or those of the publisher, the editors and the reviewers. Any product that may be evaluated in this article, or claim that may be made by its manufacturer, is not guaranteed or endorsed by the publisher.
